# Testing the Enemies Hypothesis in Peach Orchards in Two Different Geographic Areas in Eastern China: The Role of Ground Cover Vegetation

**DOI:** 10.1371/journal.pone.0099850

**Published:** 2014-06-25

**Authors:** Nian-Feng Wan, Xiang-Yun Ji, Jie-Xian Jiang

**Affiliations:** Eco-environment Protection Research Institute, Shanghai Academy of Agricultural Sciences, Shanghai Key Laboratory of Protected Horticultural Technology, Shanghai, China; Institute of Vegetables and Flowers, Chinese Academy of Agricultural Science, China

## Abstract

Many studies have supported the enemies hypothesis, which suggests that natural enemies are more efficient at controlling arthropod pests in polyculture than in monoculture agro-ecosystems. However, we do not yet have evidence as to whether this hypothesis holds true in peach orchards over several geographic locations. In the two different geographic areas in eastern China (Xinchang a town in the Shanghai municipality, and Hudai, a town in Jiangsu Province) during a continuous three-year (2010–2012) investigation, we sampled arthropod pests and predators in *Trifolium repens* L. and in tree canopies of peach orchards with and without the ground cover plant *T*. *repens*. No significant differences were found in the abundances of the main groups of arthropod pests and predators in *T*. *repens* between Hudai and Xinchang. The abundance, richness, Simpson's index, Shannon-Wiener index, and Pielou evenness index of canopy predators in ground cover areas increased by 85.5, 27.5, 3.5, 16.7, and 7.9% in Xinchang, and by 87.0, 27.6, 3.5, 17.0 and 8.0% in Hudai compared to those in the controls, respectively. The average abundance of Lepidoptera, Coleoptera, Homoptera, true bugs and Acarina canopy pests in ground cover areas decreased by 9.2, 10.2, 17.2, 19.5 and 14.1% in Xinchang, and decreased by 9.5, 8.2, 16.8, 20.1 and 16.6% in Hudai compared to that in control areas, respectively. Our study also found a higher density of arthropod species resources in *T*. *repens*, as some omnivorous pests and predators residing in *T*. *repens* could move between the ground cover and the orchard canopy. In conclusion, ground cover in peach orchards supported the enemies hypothesis, as indicated by the fact that ground cover *T*. *repens* promoted the abundance and diversity of predators and reduced the number of arthropod pests in tree canopies in both geographical areas.

## Introduction

The enemies hypothesis argues that natural enemies are more effective at controlling arthropod pests in diverse ecosystems than in simple ones [1]–[3]. This hypothesis has received support from studies of diversified agro-ecosystems [4]–[6], but evidence from diversified orchard ecosystems is scarce, even though there have been reports on pest management in orchards [7]–[8].

Ground cover vegetation, as an important diversified plantation model [9]–[11], has played a key role in enhancing crop yield, productivity, and stability in ecosystems [12]–[14]. Although the use of ground cover vegetation in peach orchards for pest management has been investigated [15]–[16], few such studies have evaluated the enemies hypothesis.

China has the largest peach plantings and the largest production of peaches in the world. Shanghai municipality and Jiangsu province in eastern China are the two most important peach-growing regions: both Shanghai, with an annual peach cultivation area of 1.5×10^4^ ha and peach yield of 1.8×10^8^ kg, and Jiangsu, with an annual peach cultivation area of 3.2×10^4^ ha and a peach yield of 3.5×10^8^ kg, have acquired national recognition as peach growing areas.

The use of ground cover vegetation in peach orchards began in China in the 1990s and was gradually applied nationwide. Recently, the use of a clover ground cover (*Trifolium repens* L.) was selected as a useful approach to enhance soil fertility in orchards [17]–[18], and the use of this clover has been widely applied in eastern China. However, whether its use also enhances natural enemies in pest control (the enemies hypothesis) has not been tested.

In our former study in Shanghai municipality and Jiangsu province in eastern China, we observed that the arthropod pests in *T*. *repens* mainly included Homoptera (in this paper Homoptera is a suborder of the Hemiptera insect, including Psyllidae, whitefly, cicadas, aphids, scale insects, i.e.), Lepidoptera, true bugs, Acarina and Coleoptera, while the predators in *T*. *repens* mainly included Araneida, Coleoptera, Neuroptera, Diptera and Hemiptera. In additional, we found that these species provided an excellent pool for canopy arthropods in peach orchards [19]. The outbreaks of canopy arthropod pests, mainly including Homoptera, true bugs, Coleoptera, Lepidoptera and Acarina pests, were severely suppressed by Araneida, Coleoptera, Neuroptera, Diptera and Hemiptera predators in canopy peach trees [19]–[20]. However, whether the abundances of the arthropod pests and predators in *T*. *repens* and in the peach canopy differing between the two different geographic locations in eastern China have not been examined, and whether *T*. *repens* ground cover enhances the level of control exerted by predators on the abundance of key pests in the orchard canopies has not been tested.

This study, therefore, sought to test the enemies hypothesis in peach orchards in the two different sites in eastern China. In particular, we sought to determine: (1) what effect the pool of predators and pests in *T*. *repens* played, and whether the abundance of the main groups of arthropod pests and predators in *T*. *repens* were different between the two geographical areas studied; (2) whether the abundance and diversity of predators in canopy orchards were enhanced by the use of clover ground cover and whether this difference varied between the two locations; and (3) whether the abundance of the main groups of arthropod pests in canopy orchards was inhibited by manipulation of *T*. *repens* ground cover and whether this differed between the two locations. If this hypothesis proved true, the use of ground cover to favor indigenous predators would be a more sustainable approach to controlling arthropod pests than the input of insecticides or exotic control agents in eastern China, or indeed in other agricultural areas.

## Methods

### Ethical statement

The Guoyuan village of Xinchang town in Shanghai municipality and Longyan village of Hudai town in Jiangsu province issued permission for the experiments in each location. No vertebrates were studied, as our study involved only arthropods (insects, mites and spiders).

### Study sites

This experiment was conducted in two locations: one in Xinchang town, Pudong district, Shanghai municipality of China (31.03°N, 121.41°E, elevation 4.3 m), and the other in Hudai town, Wuxi district, Jiangsu Province of China (31.34°N, 121.18°E, elevation 3.5 m). The two sites were both located in the Yangtze River delta alluvial plain, the Hudai site near Taihu Lake (the second largest freshwater lake in China), and the Xinchang site near the Yangtze River, 20 kilometers away from the first site. The sites share a similar climate, both belonging to the prevailing zone of the East Asian monsoon in the southern rim of North Asian tropics. Peach varieties were “Hujing” honey peach in Hudai and “Xinfeng” honey peach in Xinchang, both mid-season maturing varieties with 8–10 year-old trees that were 2–2.5 m high, and arranged in a 4×4 m grid.

### Treatment and management

A randomized block design was used in this experiment, and each treatment and the control were replicated three times in both Xinchang and Hudai. Thirty peach trees were sampled for each replicate to calculate the abundance and diversity of predators and the abundances of pest arthropods. Each plot was 100 m wide and 52 m long (5200 m^2^), and separated from adjacent plots by a 100 m long isolation belts. Treatment areas were seeded with a ground cover of the biennial clover *T. repens*, which was mowed twice a year to a height of about 10 cm. Control areas in the orchards were bare ground without weeds (which were pulled by hand) under the trees. Farm management and operation methods, including pest management, were identical between treatment and control plots in each orchard. Pest management in peach orchards mainly relies on physical and manual means, such as trimming of diseased and pest-infested peach branches and shoots in winter, fruit bagging in June, trapping insect pests in pheromone traps, and spraying with 4–5 Baume lime sulfur during the dormant period.

### Sampling methods

Sampling methods in peach orchards to measure densities of the predatory and pest arthropods in the plots followed those of Bi et al. [21]. Within each replicate plot (treatment and control areas), thirty adjacent peach trees with checkerboard type distribution and similar to each other in height and vigor, were selected as permanent sampling points to monitor the abundance of predators and pests. At each sampling date, each tree was sampled in each of the four cardinal directions (east, south, west and north) at three levels each (upper, middle and lower), such that each tree canopy was split into 12 zones [22]. The branch beating method was used to collect predators and pests from the canopy peach according to Simon et al. [23]. Branches in the tree canopy were struck with a rubber hose over a 25 cm dia collection funnel. Predators and pests falling from the branches were identified and counted immediately, but mobile arthropods on the exterior of the tree were first located and collected before beating branches.

Sampling to measure the densities of the predators and pests in the *T*. *repens* clover ground cover followed a “Z”-shaped sampling plan, with seven sampling sites (plots) in the area beneath each treatment in each peach orchard. Sample locations were spaced equidistant along the Z transect, at 12 m intervals. Each sampling location was a 1 m^2^ area defined by a wire frame. The arthropods in the *T*. *repens* ground cover were beaten by hand into a white porcelain tray (0.3 m×0.4 m) and were then immediately classified to species and the numbers of individuals within orders was counted.

Approximately 3–5 minutes were needed to collect the canopy arthropods and count the individuals of each species at each canopy level, choosing one representative twig (20–30 cm long) from each zone in each tree. Each twig from the base to the tip was intensively examined to count all the arthropods present. In addition, 3–5 minutes were taken to collect the arthropods in the *T*. *repens* ground cover at each of the seven sample points per treatment replicate. Any unidentified species in the orchards and in the *T*. *repens* were collected in vials of 80% alcohol, counted and labeled for later identification in the laboratory. Sampling was done ca every 10 days, from late March to early October in 2010, 2011, and 2012 (with some delays because of rain or other contingencies), for a total of nineteen sample dates per year.

### Data analysis

In most cases, we classified the arthropod pests collected in the *T*. *repens* ground cover into five main groups (Homoptera mainly including Aphididae, Aleyrodidae and Cicadellidae pests, true bugs mainly including Miridae, Coreidae, Pentatomidae and Tingidae pests, Lepidoptera mainly including Noctuidae, Pyralidae, Psychidae, Pieridae, Papilionidae and Lycaenidae pests, Coleoptera mainly including Melolonthidae, Rutelidae, Cetoniidae, Chrysomelidae, Curculionidae, Meloidae and Elateridae pests, and Acarina mainly including Tetranychidae pests) and similarly for the predators from the *T*. *repens* ground cover as Araneida, Coleoptera, Neuroptera, Diptera, or Hemiptera. The species composition of arthropod pests and predators collected from the *T*. *repens* ground cover can be found in Table S1.

Data from each canopy sample in each plot were collated and entered into a relational database with basic information on the predators (biological characteristics, feeding guild, development mode, host range, and taxonomic status) and on the samples (date, location in peach orchard, direction in tree canopy, and sampling area). Results from the thirty sampled peach trees for each replicate in each of treatment and control area over three years (2010–2012) in Xinchang and Hudai were used to calculate the diversity indices of predators in the canopy of peach orchards with or without the ground cover plant *T*. *repens*. Abundance, species richness, Simpson's index, Shannon-Wiener index, and the Pielou evenness index were used to measure the diversity of predators. We also classified the arthropod pests collected in tree canopies into five main groups as Lepidoptera, mainly including Ortricidae, Lyonetiidae, Pyralididae, Sphingidae, Carposinidae, Noctuidae, Cossidae, Limacodidae, Psychidae, Lymantridae, Saturniidae, Pieridae and Carposinidae: Coleoptera, mainly including Curculionidae, Cetoniidae, Rutelidae, Melolonthidae and Cerambycidae: Homoptera mainly including Aphididae, Cicadellidae, Cicadidae, Fulgoridae, Coccidae, Margarodidae, Diaspididae and Aleyrodidae: true bugs, mainly including Pentatomidae, Miridae, Tingidae and Coreidae and Acarina, mainly including Tetranychidae. The species composition of arthropod pests and predators in tree canopies can be found in Table S2.

Statistical analyses were performed using the Statistical Analysis Systems software (SigmaStat Statistical Software, SPSS Science, Chicago, IL, USA). Normal distribution and homocedasticity of all data were checked by the Kolmogorow-Smirnov test and Levene test, respectively. Three-factor analysis of variance with General Linear Model was adopted to analyze the diversity indices of canopy predators and the number of the main groups of canopy arthropod pests, so as to compare the interactive effects of different sites, years, and orchard types on the diversity of canopy predators and the abundance of the main arthropod pests. Two-factor (site and year) analysis of variance with General Linear Model was used to analyze the number of the main groups of arthropod pests and predators in *T. repens* ground cover. The sites were Xinchang and Hudai; the years were 2010, 2011, and 2012; the orchard types were ground cover or bare ground, and the interactive effects involved were site × year, site × orchard type, year × orchard type, and site × year × orchard type. If the values of the diversity indices of canopy predators and the abundance of canopy pests were not significantly affected by the years with the above three-factor analysis of variance, we took the three years of data as a whole to compare the differences among means related to treatment and control in Xinchang and in Hudai, respectively, with Tukey's Honestly Significant Difference (HSD) test at the 0.05 level. If the values of the abundance of the main groups of arthropod pests and predators in *T*. *repens* ground cover plots were not significantly affected by the years with the above two-factor analysis of variance, we again took the three years of data as a whole to compare the differences among means between Xinchang and Hudai, respectively, with independent samples *t*-test at the 0.05 level.

## Results

### Effect of ground cover on the abundance of the main groups of arthropod pests and predators in the *Trifolium repens* clover ground cover

The factors of site and year had no significant effects on the abundance of arthropod pests of Lepidoptera, Homoptera, true bugs, Acarina or Coleoptera, and the same effects were indicated by the interactions of the two factors (Table 1). Neither the single factors (site and year) nor the interaction of two factors (site × year) significantly affected the abundance of predators of Araneida, Coleoptera, Neuroptera, Diptera or true bugs (Table 2).

**Table 1:** Variance analysis of different sites and years against the abundance of the main groups of arthropod pests in *Trifolium repens* L.

Treatment	Lepidoptera		Homoptera		True bug		Acarina		Coleoptera	
	*F*	Sig.	*F*	Sig.	*F*	Sig.	*F*	Sig.	*F*	Sig.
Site	0.764	0.384	1.983	0.162	0.853	0.358	0.510	0.476	0972	0326
Year	0.426	0.654	1.103	0.336	1.483	0.231	1.302	0.276	0522	0595
Site × year	0.037	0.964	0.295	0.745	0.372	0.690	0.091	0.913	2.029	0136

*Notes*: here Homoptera is a suborder of the Hemiptera insect, including Psyllidae, whitefly, cicadas, aphids, scale insects, i.e.; in *Trifolium repens* L. Lepidoptera pests mainly included Noctuidae, Pyralidae, Psychidae, Pieridae, Papilionidae and Lycaenidae; Homoptera pests mainly included Aphididae, Aleyrodidae and Cicadellidae; true bugs mainly included Miridae, Coreidae, Pentatomidae and Tingidae pests; Coleoptera pests mainly included Melolonthidae, Rutelidae, Cetoniidae, Chrysomelidae, Curculionidae, Meloidae and Elateridae; Acarina mainly included Tetranychidae. The same below.

**Table 2:** Variance analysis of different sites and years against the abundance of the main groups of arthropod predators in *T*. *repens*.

Treatment	Araneida		Coleoptera		Neuroptera		Diptera		Hemiptera	
	*F*	Sig.	*F*	Sig.	*F*	*F*	Sig.	Sig.	*F*	Sig.
Site	1.482	0.226	1.604	0.208	0.242	0.624	3.676	0.058	2.036	0.156
Year	0.291	0.748	1.241	0.293	0.007	0.994	1.747	0.179	2.205	0.115
Site × year	0.053	0.948	0.028	0.972	0.868	0.423	0.883	0.416	1.295	0.278

*Notes*: in *T*. *repens* Araneida predators mainly included Thomisidae, Erigonidae, Salticidae, Araneidae, Lycosidae, Thomisidae, Tetragnathidae, Dictynidae, Theridiidae and Oxyopidae; Coleoptera predators mainly included Coccinellidae and Carabidae; Neuroptera predators mainly included Chrysopidae; Diptera predators mainly included Syrphidae and Tachinidae; Hemiptera predators mainly included Anthocoridae. The same below.

The abundances of Lepidoptera, Homoptera, true bugs, and Acarina arthropod pests in plots with *T*. *repens* ground cover in Xinchang were all lower, while that of Coleoptera was slightly higher, than that in Hudai, but these differences were not significant (*t*-test: Homoptera, *t* = 1.416, df = 112, *P* = 0.603; Lepidoptera, *t* = 0.886, df = 112, *P* = 0.377; true bugs, *t* = 0.925, df = 112, *P* = 0.986; Acarina, *t* = 0.718, df = 112, *P* = 0.157; Coleoptera, *t* = 0.981, df = 112, *P* = 0.759) (Table 3).

**Table 3:** Comparison of the abundance of the main groups of arthropod pests in *T*. *repens*.

Site	Lepidoptera	Homoptera	True bug	Acarina	Coleoptera
Xinchang, Shanghai	14.93±1.51a	14.73±0.88a	6.74±0.36a	23.74±2.76a	3.58±0.09a
Hudai, Jiangsu	16.95±1.71a	16.44±0.83a	7.23±0.39a	26.71±3.08a	3.46±0.08a

*Notes*: here Homoptera is a suborder of the Hemiptera insect, including Psyllidae, whitefly, cicadas, aphids, scale insects, i.e.; the same letters in the same column indicate that the means are not significantly different at *P*<0.05 (independent samples *t*-test) between Xinchang and Hudai during the three years (57 sample dates were considered as 57 replicates). Means of five groups of arthropod pests were calculated from a sample size of one square meter of *T*. *repens*.

While the numbers of Araneida, Coleopteran and Hemipteran predators were all higher, and the numbers of Neuropteran and Dipteran predators in plots with *T*. *repens* ground cover were both lower in Xinchang than in Hudai, these differences were not significant (*t*-test: Araneida, *t* = 1.236, df = 112, *P* = 0.072; Coleoptera, *t* = 1.275, df = 112, *P* = 0.319; Neuroptera, df = 112, *t* = 0.497, *P* = 0.918; Diptera, *t* = 1.907, df = 112, *P* = 0.616; Hemiptera *t* = 1.408, df = 112, *P* = 0.160) (Table 4).

**Table 4:** Comparison on the abundance of the main groups of predators in *T*. *repens*.

Site	Araneida	Coleoptera	Neuroptera	Diptera	Hemiptera
Xinchang, Shanghai	21.77±1.60a	17.65±1.35a	6.81±0.38a	3.22±0.11a	4.22±0.18a
Hudai, Jiangsu	19.18±1.36a	15.33±1.22a	7.08±0.38a	3.51±0.11a	3.90±0.15a

*Notes*: the same letters in the same column indicate that the means are not significantly different at *P*<0.05 (independent samples *t*-test) between Xinchang and Hudai during the three years (57 sample dates were considered as 57 replicates). Means of five groups of predators were calculated from a sample size of one square meter of *T*. *repens*.

**Figure 1:**
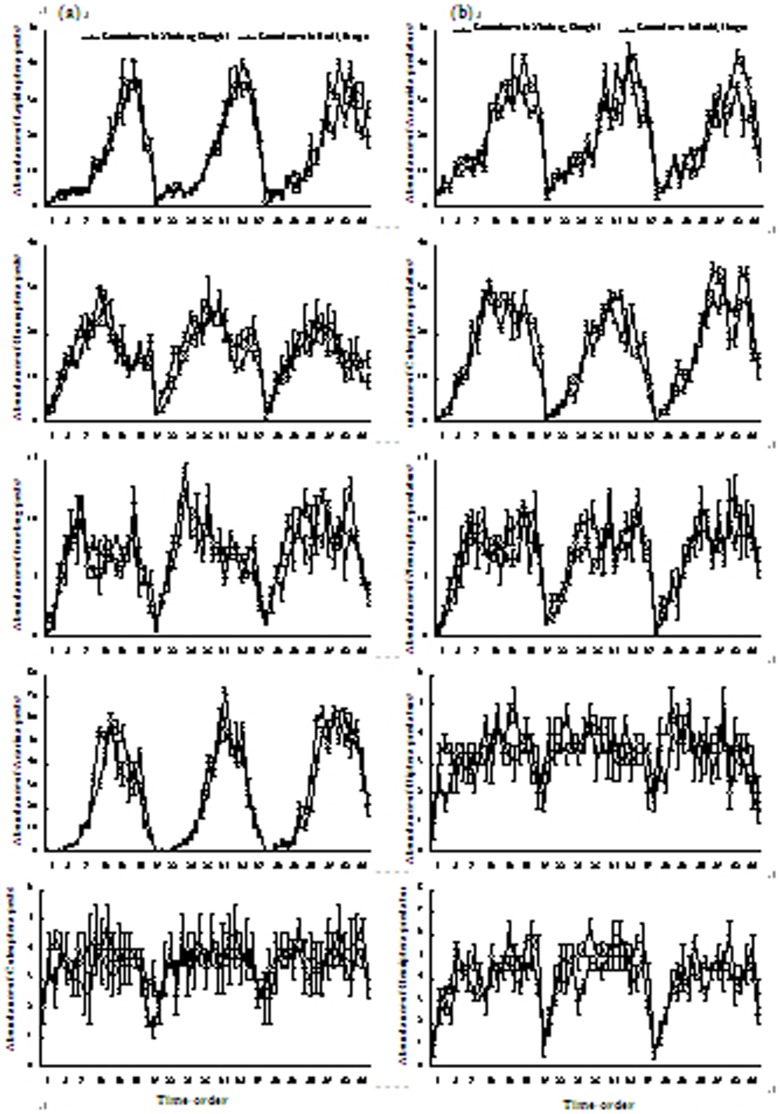
Dynamics of the abundance of the main groups of arthropod pests (a) and arthropod predators (b) in plots with *Trifolium repens* L. ground cover from a sample size of 1 m^2^ in peach orchards. Vertical bars denote SE. The numbers on the *X*-axis indicate the sampling times, i.e., the first 19 times (1 to 19) were conducted from late-March to early-October 2010, the second 19 times (20 to 38) from late-March to early-October 2011, and the third 19 times (39 to 57) from late-March to early-October 2012.

The dynamics of the abundance of the main groups of arthropod pests and predators in plots with *T*. *repens* ground cover were similar for Xinchang and Hudai. In both sites, the abundances of pest Homoptera, Lepidoptera, true bugs, Acarina, and Coleoptera, as well as that of the predacious Araneida, Coleoptera, Neuroptera, Diptera, and Hemiptera increased during spring but stabilized thereafter (Figure 1).

### Effect of ground cover on the diversity of canopy predators

The single factors of site and year had no significant effect on the diversity indices of canopy predator communities, while the factor of orchard type did have a significant effect. The interactive effects of two factors (site × year, site × orchard type, and year × orchard type) and of all three factors (site × year × orchard type) on the diversity indices were also not significant, respectively (Table 5).

**Table 5:** Variance analysis of different sites, years and orchard types against the diversity indices of canopy predators in peach orchards.

Treatment	Abundance		Richness		Simpson's index		Shannon-Wiener index		Pielou evenness index	
	*F*	Sig.	*F*	Sig.	*F*	Sig.	*F*	Sig.	*F*	Sig.
Site	0.145	0.704	0.145	0.703	0.060	0.807	0.250	0.618	0.012	0.912
Year	0.132	0.876	0.707	0.494	2.854	0.060	2.450	0.089	2.757	0.066
Orchard type	92.282	<0.001	435.572	<0.001	728.062	<0.001	903.413	<0.001	2.052×10^3^	<0.001
Site × year	0.103	0.902	0.063	0.939	0.285	0.752	0.038	0.963	1.211	0.300
Site × orchard type	0.003	0.958	0.034	0.853	0.048	0.828	0.025	0.875	0.037	0.848
Year × orchard type	0.583	0.559	0.114	0.892	1.221	0.297	0.165	0.848	0.683	0.506
Site × year × orchard type	0.058	0.943	0.023	0.977	0.250	0.779	0.181	0.834	0.869	0.421

The abundance, richness, Simpson's index, Shannon-Wiener index, and Pielou evenness index of predator communities in ground cover areas were all significantly greater than those in the control areas in both Xinchang and Hudai (abundance: *F*_3, 224_ = 31.694, *P*<0.001; richness: *F*_3, 224_ = 149.374, *P*<0.001; Simpson's index: *F*_3, 224_ = 241.408, *P*<0.001; Shannon-Wiener index: *F*_3, 224_ = 304.400, *P*<0.001; Evenness: *F*_3, 224_ = 674.945, *P*<0.001) (Table 6).

**Table 6:** Comparison of diversity indices of canopy predators in peach orchards with and without ground cover by *T*. *repens* (means ± SE).

Orchard type (site)	Abundance	Richness	Simpson's index	Shannon-Wiener index	Pielou evenness index
Ground cover (Xinchang, Shanghai)	315.0±16.8a	26.0±0.2a	0.9645±0.0003a	4.6716±0.0094a	0.9932±0.0005a
Bare ground (Xinchang, Shanghai)	169.8±10.2b	20.4±0.3b	0.9319±0.0016b	4.0025±0.0245b	0.9201±0.0021b
Ground cover (Hudai, Jiangsu)	308.4±19.6a	25.9±0.2a	0.9650±0.0004a	4.6640±0.0117a	0.9933±0.0005a
Bare ground (Hudai, Jiangsu)	164.9±10.3b	20.3±0.4b	0.9320±0.0018b	3.9878±0.0340b	0.9196±0.0024b

*Notes*: Different letters in the same column indicate that the means are significantly different at *P*<0.05 (*HSD* test) within groups of ground cover (treatment) and bare ground (control) in Xinchang and Hudai during the three years (57 sample dates were considered as 57 replicates). Means of five diversity indices were calculated from a total of 30 trees per treatment or control.

**Figure 2:**
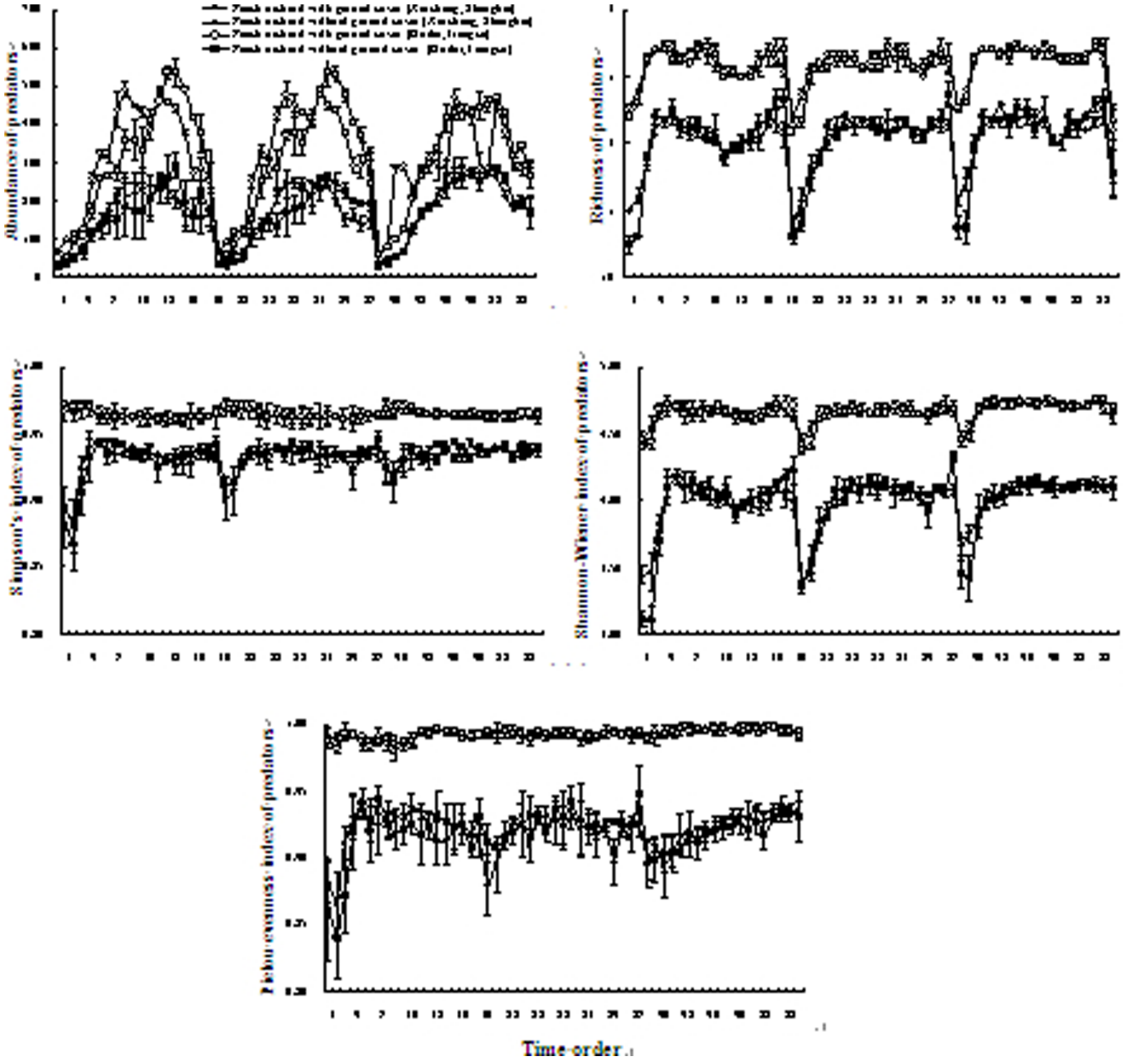
Dynamics of the diversity indices of predatory canopy arthropod in peach orchards with and without ground cover of *T*. *repens*. Vertical bars denote SE. The numbers on the *X*-axis indicate the sampling times, i.e., the first 19 times (1 to 19) were conducted from late-March to early-October 2010, the second 19 times (20 to 38) from late-March to early-October 2011, and the third 19 times (39 to 57) from late-March to early-October 2012.

The five diversity indices of canopy predator communities were consistently higher in ground cover areas than control areas. Irrespective of ground cover, all diversity indices increased during spring but stabilized thereafter in both sites (Figure 2).

### Effect of ground cover on the abundance of the main groups of canopy arthropod pests

Ground covers of *T*. *repens* in peach orchards had significant effects on the numbers of pest Coleoptera, Homoptera, and true bugs, but no significant effects on the numbers of pest Lepidoptera or Acarina. The single factor of site had a significant effect on the number of Coleoptera pests, but no significant effect on the number of the other four groups of pests, while the single factor of year had no significant effect on the numbers of pests in any of the five groups. Similarly, none of the interactive factors (site × year, site × orchard type, year × orchard type, and site × year × orchard type) had any significant effects on the numbers of pests in any of the five groups (Table 7).

**Table 7:** Variance analysis of different sites, years and orchard types against the abundance of the main groups of canopy arthropod pests in peach orchards.

Treatment	Lepidoptera		Coleoptera		Homoptera		True bug		Acarina	
	*F*	Sig.	*F*	Sig.	*F*	Sig.	*F*	Sig.	*F*	Sig.
Site	0.233	0.630	12.955	<0.001	0.350	0.555	0.158	0.691	0.288	0.592
Year	0.311	0.733	0.228	0.797	0.015	0.986	0.419	0.659	0.349	0.706
Orchard type	1.218	0.271	55.040	<0.001	6.090	0.014	7.227	0.008	2.477	0.117
Site × year	0.678	0.509	0.636	0.530	0.323	0.725	0.011	0.989	0.043	0.958
Site × orchard type	<0.001	0.998	1.036	0.310	0.007	0.932	0.007	0.935	0.035	0.851
Year × orchard type	0.040	0.961	0.512	0.600	0.028	0.972	0.136	0.873	0.178	0.837
Site × year × orchard type	0.084	0.919	0.133	0.875	0.049	0.952	0.004	0.996	0.031	0.969

*Notes*: here Homoptera is a suborder of the Hemiptera insect, including Psyllidae, whitefly, cicadas, aphids, scale insects, i.e.; in tree canopies Lepidoptera pests mainly included Ortricidae, Lyonetiidae, Pyralididae, Sphingidae, Carposinidae, Noctuidae, Cossidae, Limacodidae, Psychidae, Lymantridae, Saturniidae, Pieridae and Carposinidae, Coleoptera pests mainly included Curculionidae, Cetoniidae, Rutelidae, Melolonthidae and Cerambycidae, Homoptera pests mainly included Aphididae, Cicadellidae, Cicadidae, Fulgoridae, Coccidae, Margarodidae, Diaspididae and Aleyrodidae, True bug pests mainly included Pentatomidae, Miridae, Tingidae and Coreidae, and Acarina mainly included Tetranychidae.

In neither Xinchang nor Hudai did ground cover have any significant effect on the number of pests in any of the groups except Coleoptera (one-way ANOVA: Lepidoptera, *F*_3, 224_ = 0.496, *P* = 0.685; Coleoptera, *F*_3, 224_ = 23.533, *P*<0.001; Homoptera, *F*_3, 224_ = 2.220, *P* = 0.087; true bugs, *F*_3, 224_ = 2.542, *P* = 0.057; Acarina, *F*_3, 224_ = 0.963, *P* = 0.411) (Table 8). However, compared to those in control areas, the numbers of pest Lepidoptera, Coleoptera, Homoptera, true bugs, and Acarina in ground cover areas decreased by 9.2, 10.2, 17.2, 19.5 and 14.1%, respectively, in Xinchang and by 9.5, 8.2, 16.8, 20.1, and 16.6%, respectively, in Hudai.

**Table 8:** Comparison on the abundance of the main groups of canopy arthropod pests in peach orchards with ground cover and without ground cover.

Orchard type (site)	Lepidoptera	Coleoptera	Homoptera	True bug	Acarina
Ground cover (Xinchang, Shanghai)	161.6±15.1a	41.27±0.50c	115.9±8.6a	86.3±6.8a	110.1±11.9a
Bare ground (Xinchang, Shanghai)	177.9±16.2a	45.98±0.61a	140.0±11.0a	107.2±8.1a	128.1±13.3a
Ground cover(Hudai, Jiangsu)	154.5±12.8a	39.84±0.54c	111.1±8.3a	88.8±7.4a	114.7±11.8a
Bare ground (Hudai, Jiangsu)	170.7±13.7a	43.40±0.55b	133.6±8.9a	111.1±9.0a	137.5±13.9a

*Notes*: here Homoptera is a suborder of the Hemiptera insect, including Psyllidae, whitefly, cicadas, aphids, scale insects, i.e.; different letters in the same column indicate that the means are significantly different at *P*<0.05 (*HSD* test) within groups of ground cover (treatment) and bare ground (control) in Xinchang and Hudai during the three years (57 sample dates were considered as 57 replicates). Means of the five orders of arthropod pests were calculated from a total 30 trees per treatment or control.

Meanwhile, there were no significant differences in the number of Lepidoptera, Coleoptera, Homoptera, true bug, or Acarina pests between Xinchang and Hudai in ground cover areas (HSD: Xinchang vs. Hudai: Lepidoptera, *P* = 0.986; Coleoptera, *P* = 0.255; Homoptera, *P* = 0.984; true bug, *P* = 0.996; Acarina, *P* = 0.994). However, in control areas, while the difference in the number of Lepidoptera, Homoptera, true bugs, or Acarina pests between Xinchang and Hudai was not significant, the difference in the number of Coleoptera was significant (HSD: Xinchang vs. Hudai: Lepidoptera, *P* = 0.986; Coleoptera, *P* = 0.006; Homoptera, *P* = 0.962; true bug, *P* = 0.986; Acarina, *P* = 0.954) (Table 8).

**Figure 3:**
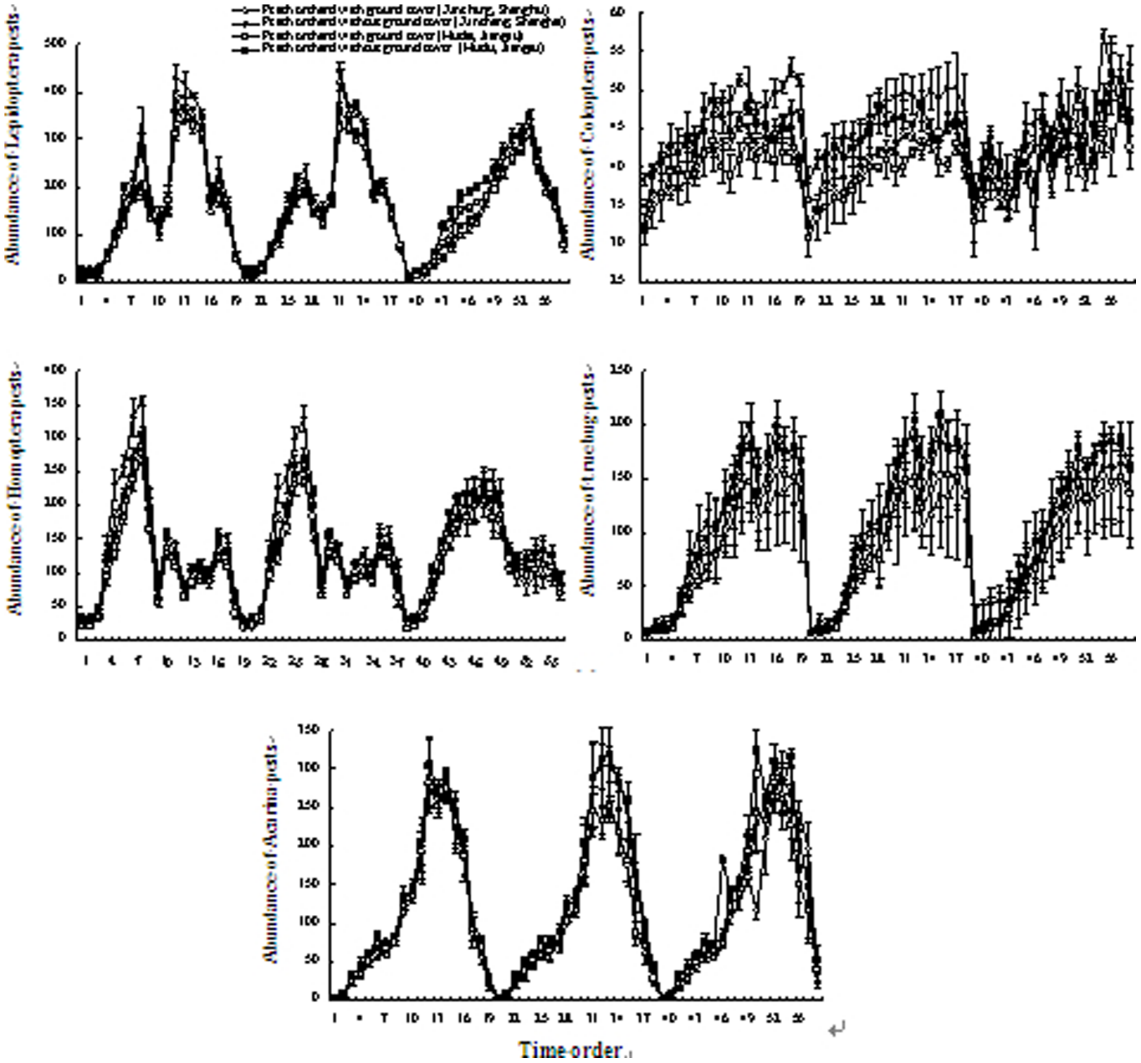
Dynamics of the five-group arthropod pests in peach orchards with and without ground cover of *T*. *repens*. Vertical bars denote SE. The numbers on the *X*-axis indicate the sampling times, i.e., the first 19 times (1 to 19) were conducted from late-March to early-October 2010, the second 19 times (20 to 38) from late-March to early-October 2011, and the third 19 times (39 to 57) from late-March to early-October 2012.

The abundances of pests of all five groups of arthropods were consistently lower in ground cover than control areas. Irrespective of ground cover, the abundances of Coleoptera and true bugs increased during spring but stabilized thereafter, while the abundance of Acarina pests was relatively higher in the 7th–9th month with higher temperature, the abundance of Homoptera pests was highest in June, and the abundance of Lepidoptera pests was highest in August at both sites (Figure 3).

## Discussion

Root [24] proposed the enemies hypothesis, which states that predators are more abundant and effective in diverse systems than in simple ones. Andow [4] maintained that diversified vegetational ecosystems would support the enemies hypothesis, as both the richness and abundance of predators would increase, as was found in ecosystems with ground covers [13], [15], [25]. On the other hand, some experiments have indicated that predators are not significantly influenced by cropping pattern [26]–[27], and some results do not support the enemies hypothesis [28]–[30].

Our results indicated that when the ground beneath peach orchards was covered with *T*. *repens*, the richness and abundance of canopy predators increased by 27.5 and 85.5% in Xinchang and by 27.6 and 87.0% in Hudai, respectively. Similar studies found that the richness and abundance of predators increased by ground cover in pecan orchards [31], apple orchards [32], and lemon trees [33], as well as by grass cover (*Elymus trachycaulus*) in maize fields [34].

Ground cover vegetation can create a suitable ecological structure within the agricultural landscape to provide food resources for predators to feed and reproduce [35]–[36]. Our study found the average abundance of the main predators in *T*. *repens* ground cover in Xinchang and in Hudai to be 53.7 and 49.0 individuals per m^2^, respectively, which provided a strong pool of predators potentially able to forage in tree canopies in peach orchards. The principal value of ground cover in orchards might be the movement of predators from the orchard floor to the trees, thus augmenting the richness and abundance of predators in trees. We observed, for instance, that predators such as spiders, ladybirds, lacewings, carabid beetles, and hoverflies could readily disperse from *T*. *repens* ground cover to the peach tree canopy, which might explain the higher diversity of predators in the orchard canopy.

In addition, ground cover vegetation could also offer refuge for predators avoiding adverse influences from alien disturbance to ecosystems [37]. Similar research found that uncut strips of lucerne (*Medicago sativa*) provided refuge to a range of coccinellid and hemipteran predators [38]. In practice, we observed that predators always actively transferred into the *T*. *repens* ground cover to avoid man-made disturbance when pathogen-infested peach branches or shoots were trimmed, and that adverse meteorological conditions (such as an abrupt drops of temperatures or severe rain) likewise caused predators to take shelter in the ground cover stratum.

Some omnivorous arthropod pests resident in *T*. *repens* ground cover, such as harmful mites, noctuids, and whitefly *Bemisia tabaci* (Gennadius) (the average abundance of the three harmful groups was 23.7, 5.1 and 5.8 per m^2^ in Xinchang, and 26.7, 5.2 and 4.9 per m^2^ in Hudai, respectively), could transfer from *T*. *repens* to peach trees for feeding. Similarly, Gruys [39] reports that tarnished plant bug could disperse from cover crops to damage field, orchard, and row crops, and Tedders et al. [40] notes that *Tetranychus urticae* Koch moved from clover into pecan trees.

*T*. *repens* ground cover enhanced the ecological functions of predators to control pests in peach orchards [41]. In peach orchards, hoverflies mainly prey on aphids, scale insects and leafhoppers, while spiders, ladybirds and lacewings mainly prey on aphids, leafhoppers and bugs. Our study indicated that ground cover promoted the biological control service function as the abundance of canopy spiders, ladybirds, lacewings and hoverflies increased by 38.1%, 81.5%, 93.0% and 80.2% in Xinchang and by 43.8%, 91.2%, 126.7% and 117.1% in Hudai, respectively.

Among the main groups of arthropod pests, the aphids which make peach leaves curl and the oriental fruit moth (*Grapholitha molesta* (Busck)) which makes peach shoots wither are the most serious insect pests in eastern China [41]. Compared to bare ground plots, the abundances of aphids and *G*. *molesta* decreased respectively by more than 30% both in Shanghai and Jiangsu. Meanwhile, the rate of pests bored into the peach sarcocarp (a typical indicator of fruit quality) was decreased by more than 8% and the peach yield was increased by more than 5% in both sites.

While geographical distribution might have a certain influence on species diversity and abundance [42], our study found that the richness of arthropod pests and predators in *T*. *repens* in the two sites was very similar, with 44 species of arthropod pests belonging to 26 families (8 orders) and 27 species of predators belonging to 16 families (5 orders) in Xinchang, and 45 species of arthropod pests belonging to 27 families (8 orders) and 29 species of predators belonging to 16 families (5 orders) in Hudai. The abundance of the main groups of arthropod pests and predators in *T*. *repens* was likewise not significantly affected by the two geographical locations. A more comprehensive, long-term and large-scale geographical distribution investigation of the potential ground cover vegetation in peach orchards is the logical next area of study.

## Supporting Information

Table S1 **The species composition of arthropod pest and predatory arthropod in** ***T*** **.** ***repens*** **in Xinchang (31.03°N, 121.41°E, elevation 4.3 m) and in Hudai (31.34°N, 121.18°E, elevation 3.5 m).**
(DOC)

Table S2 **The species composition of arthropod pests and predatory arthropods in peach orchards with and without ground cover** ***T*** **.** ***repens*** **in Xinchang (31.03°N, 121.41°E, elevation 4.3 m) and in Hudai (31.34°N, 121.18°E, elevation 3.5 m).**
(DOC)
