# Supplementary material for: Testing the Enemies Hypothesis in Peach Orchards in Two Different Geographic Areas in Eastern China: The Role of Ground Cover Vegetation
Source: PLoS One. 2014 Jun 25;9(6):e99850. doi: 10.1371/journal.pone.0099850 (PMC4070891; doi:10.1371/journal.pone.0099850)
Supplement: Table S1 — The species composition of arthropod pest and predatory arthropod in T . repens in Xinchang (31.03°N, 121.41°E, elevation 4.3 m) and in Hudai (31.34°N, 121.18°E, elevation 3.5 m). (DOC) [file pone.0099850.s001.doc]

**Table S1 The species composition of arthropod pest and predatory arthropod in *T*. *repens* in Xinchang (31.03°N, 121.41°E, elevation 4.3 m) and in Hudai (31.34°N, 121.18°E, elevation 3.5 m)**

| Functional group | Group | Family | Species | |
| --- | --- | --- | --- | --- |
|  |  |  | Xinchang, Shanghai | Hudai, Jiangsu province |
| Arthropod  pest | Coleoptera | Melolonthidae | *Holotrichia parallela*, *Maladera orientalis* | *H*. *parallela*, *M*. *orientalis* |
|  |  | Rutelidae | *Anomala corpulenta* | *A*. *corpulenta* |
|  |  | Cetoniidae | *Oxycetonia sp.* | *O*. *sp.* |
|  |  | Chrysomelidae | *Phyllotreta striolata* Fabricius*, Colaphellus sp.* | *P*. *striolata, C*. *sp.* |
|  |  | Curculionidae | *Hypera postica* Gyllenhai, *Sympiezomias sp.* | *H*. *postica*, *S*. *sp.* |
|  |  | Meloidae | *Epicauta sp.* | *E*. *sp.* |
|  |  | Elateridae | *Pleonomus canaliculatus* | *P*. *canaliculatus* |
|  | Lepidoptera | Noctuidae | *Spodoptera litura*, *Spodoptera exigua*, *Spodoptera sp. Sidemia depravata*, *Agrotis ypsilon*, *Agrotis sp.*, *Pseudaletia unipuncta* | *S*. *litura*, *S*. *exigua*, *S*. *depravata*, *A*. *ypsilon*, *A*. *sp.*, *P*. *unipuncta* |
|  |  | Pyralidae | *Loxostege sticticalis*, *Hymenia sp.* | *L*. *sticticalis*, *H*. *sp.* |
|  |  | Psychidae | *Cryptothelea minuscala* | *C*. *minuscala* |
|  |  | Pieridae | *Pieris rapae* | *P*. *rapae* |
|  |  | Papilionidae | *Papilio sp*. | *P*. *sp*. |
|  |  | Lycaenidae | *Aricia sp*. | *A*. *sp*. |
|  | Homoptera | Aphididae | *Aphis medicagini* | *A*. *medicagini* |
|  |  | Aleyrodidae | *Bemisia tabaci* (Gennadius) | *B*. *tabaci* (Gennadius) |
|  |  | Cicadellidae | *Cicadella viridis*, *Empoasca sp*. | *C*. *viridis*, *E*. *sp*. |
|  | True bug | Miridae | *Adelphocoris fasciaticollis*, *Lygus lucorum*, *Adelphocoris lineolatus* | *A*. *fasciaticollis*, *L*. *lucorum*, *A*. *lineolatus* |
|  |  | Coreidae | — | *Riptortus sp.* |
|  |  | Pentatomidae | *Halyomorpha picus*, *Dolycoris baccarum* | *H*. *picus*, *D*. *baccarum* |
|  |  | Tingidae | *Stephanitis sp.* | *S*. *sp.* |
|  | Acarina | Tetranychidae | *Tetranychus cinabarinus* Boisdu, *Tetranychus urticae* | *T*. *cinabarinus*, *T*. *urticae* |
|  | Orthoptera | Acrididae | *Atractomorpha sinensis*, *Acrida cinerea* | *A*. *sinensis*, *A*. *cinerea* |
|  |  | Tettigoniidae | *Tettigonia chinensis* Willemse | *T*. *chinensis* |
|  |  | Gryllidea | *Gryllus sp.* | *G*. *sp.* |
|  |  | Gryllotalpidae | *Gryllotalpa orientalis* Burmeister | *G*. *orientalis* |
|  | Thysanoptera | Thripidae | *Frankliniella occidentalis*, *Frankliniella sp*., *Thrips sp*. | *F*. *occidentalis*, *F*. *sp*., *T. sp*. |
|  | Diptera | Agromyzidae | *Liriomyza trifolii* | *L*. *trifolii* |
| Predatory  arthropod | Araneae | Thomisidae | *Misumenops tricuspidatus, Thomisus* sp*.* | *M*. *tricuspidatus, T*. sp*.* |
|  |  | Erigonidae | *Erigonidium graminicola* | *E*. *graminicola* |
|  |  | Salticidae | *Myrmarachne* sp*.* | *M.* sp. |
|  |  | Araneidae | *Argiope amoena*, *Neoscon,doenitzi*, *Argiope bruennichi*, *Araneus ventricosus* | *A*. *amoena*, *N*.*,doenitzi*, *A*. *bruennichi*, *A*. *ventricosus*, *Neoscona* sp*.*, *Neoscona* spp*.* |
|  |  | Lycosidae | *Lycosa pseudoamulata*, *Lycosa* sp. | *L*. *pseudoamulata*, *L*. *pseudoamulata* |
|  |  | Thomisidae | *Misumenopos tricuspidata* (Fahricius) | *M*. *tricuspidata* |
|  |  | Tetragnathidae | *Tetragnatha* sp. | *T*. sp. |
|  |  | Dictynidae | *Dictyna felis* Boes. et Str. | *D*. *felis* |
|  |  | Theridiidae | *Theridion octomaculatum* Boes. et Str. | *T*. *octomaculatum* |
|  |  | Oxyopidae | *Oxyopes sertatus* | *O*. *sertatus* |
|  | Coleoptera | Coccinellidae | *Leis axyridis* (Pallas), *Propylaea japonica* Thunbery, *Chilocorus kuwanae* Silvestri, *Coccinella septempunctata* | *L*. *axyridis*, *P*. *japonica*, *C*. *kuwanae*, *C*. *septempunctata* |
|  |  | Carabidae | *Carabus sp.* | *C*. *sp.* |
|  | Neuroptera | Chrysopidae | *Chrysopa sinica* Tieder, *Sympetrum Croceolum*, *Chrysopa formosa* Brauer | *C*. *sinica*, *S*. *Croceolum*, *C*. *formosa* |
|  | Diptera | Syrphidae | *Metasyrphus* sp. | *M.* sp. |
|  |  | Tachinidae | *Compsilura* sp*.* | *C*. sp*.* |
|  | Hemiptera | Anthocoridae | *Oriu* sp. | *O*. *minuius*, *Oriu* sp. |

*Notes:* “－” means that there’s no corresponding species.
