# Supplementary material for: Testing the Enemies Hypothesis in Peach Orchards in Two Different Geographic Areas in Eastern China: The Role of Ground Cover Vegetation
Source: PLoS One. 2014 Jun 25;9(6):e99850. doi: 10.1371/journal.pone.0099850 (PMC4070891; doi:10.1371/journal.pone.0099850)
Supplement: Table S2 — The species composition of arthropod pests and predatory arthropods in peach orchards with and without ground cover T . repens in Xinchang (31.03°N, 121.41°E, elevation 4.3 m) and in Hudai (31.34°N, 121.18°E, elevation 3.5 m). (DOC) [file pone.0099850.s002.doc]

**Appendix S2** The species composition of arthropod pests and predatory arthropods in peach orchards with and without ground cover *T*. *repens* in Xinchang (31.03°N, 121.41°E, elevation 4.3 m) and in Hudai (31.34°N, 121.18°E, elevation 3.5 m)

| Functional group | Group | Family | Species | |
| --- | --- | --- | --- | --- |
|  |  |  | Xinchang, Shanghai | Hudai, Jiangsu province |
|  |  |  | Ground cover (bare ground) | Ground cover (bare ground) |
| Arthropod pests | Lepidoptera | Ortricidae | *Adoxophyes orana*, *Acleris fimbriana*, *Grapholitha molesta* (Busck) (*A*. *orana*, *A*. *fimbriana*, *G*. *molesta*) | *A*. *orana*, *A*. *fimbriana*, *G*. *molesta* (*A*. *orana*, *A*. *fimbriana*, *G*. *molesta*) |
|  |  | Lyonetiidae | *Lyonetia clerkella* L. (*L*. *clerkella*) | *L*. *clerkella* (*L*. *clerkella*) |
|  |  | Pyralididae | *Conogethes punctiferalis* (*C*. *punctiferalis*) | *C*. *punctiferalis* (*C*. *punctiferalis*) |
|  |  | Sphingidae | *Marumba gaschkewitschii* Bremer et Grey (*M*. *gaschkewitschii*) | *M*. *gaschkewitschii* (*M*. *gaschkewitschii*) |
|  |  | Carposinidae | *Carposina nipponensis* (*C*. *nipponensis*) | *C*. *nipponensis* (*C*. *nipponensis*) |
|  |  | Noctuidae | *Spodoptera litura* Fabricius, *Spodoptera exigua* Hübner (－) | *S*. *litura*, *S*. *exigua* (－) |
|  |  | Cossidae | *Cossus* sp*.* (*Cossus* sp*.*) | *Cossus* sp*.* (*Cossus* sp*.*) |
|  |  | Limacodidae | *Latoia consocia* Walker, *Latoia lepida* (Cramer), *Cnidocampa flavescens* (Walker), *Setora postornata* (Hampson) (*L*. *consocia*, *L*. *lepida*, *C*. *flavescens*, *S*. *postornata*) | *L*. *consocia*, *L*. *lepida*, *C*. *flavescens*, *S*. *postornata* (*L*. *consocia*, *L*. *lepida*, *C*. *flavescens*, *S*. *postornata*) |
|  |  | Psychidae | *Cryptothelea minuscala* Butler (*C*. *minuscala*) | *C*. *minuscala* (*C*. *minuscala*) |
|  |  | Lymantridae | *Euproctis similis* Fueezssly, *Stilprotia salicis* (Linnaeus) (*E*. *similis*, *S*. *salicis*) | *E*. *similis*, *S*. *salicis* (*E*. *similis*, *S*. *salicis*) |
|  |  | Saturniidae | *Actias ningpoana* Fielder (*A*. *ningpoana*) | *A*. *ningpoana* (*A*. *ningpoana*) |
|  |  | Pieridae | *Pieris rapae* (－) | *P*. *rapae* (－) |
|  |  | Carposinidae | *Carposina nipponensis* (*C*. *nipponensis*) | *C*. *nipponensis* (*C*. *nipponensis*) |
|  | Coleoptera | Curculionidae | *Sympiezomias velatus* (*S*. *velatus*) | *S*. *velatus* (*S*. *velatus*) |
|  |  | Cetoniidae | *Oxycetonia jucunda* Faldermann, *Protaetia brevitarsis* Lewis (*O*. *jucunda*, *P*. *brevitarsis*) | *O*. *jucunda*, *P*. *brevitarsis* (*O*. *jucunda*, *P*. *brevitarsis*) |
|  |  | Rutelidae | *Anomala corpulenta* Motschulsky (*A*. *corpulenta*) | *A*. *corpulenta* (*A*. *corpulenta*) |
|  |  | Melolonthidae | *Holotrichia parallela* Motschulsky, *Maladera orientalis* Motschulsky (*H*. *parallela*, *M*. *orientalis*) | *H*. *parallela*, *M*. *orientalis* (*H*. *parallela*, *M*. *orientalis*) |
|  |  | Cerambycidae | *Aromia bungii* Faldermann (*A. bungii*) | *A. bungii* (*A. bungii*) |
|  | Homoptera | Aphididae | *Myzus persicae* (Sulzer), *Hyalopterus arundimis* Fabricius, *Myzus momonis* (*M*. *persicae*, *H*. *arundimis*, *M*. *momonis*) | *M*. *persicae*, *H*. *arundimis*, *M*. *momonis* (*M*. *persicae*, *H*. *arundimis*, *M*. *momonis*) |
|  |  | Cicadellidae | *Empoasca flavescens*, *Erythroneura sudra* (*E*. *flavescens*, *E*. *sudra*) | *E*. *flavescens*, *E*. *sudra* (*E*. *flavescens*, *E*. *sudra*) |
|  |  | Cicadidae | *Cryptotympana atrata* (*C*. *atrata*) | *C*. *atrata* (*C*. *atrata*) |
|  |  | Fulgoridae | *Euricania ocellus*, *Ricania speculum* (Walker), *Lycorma delicatula* (White) (*E*. *ocellus*, *R*. *speculum*) | *E*. *ocellus*, *R*. *speculum*, *L*. *delicatula* (*E*. *ocellus*, *R*. *speculum*, *L*. *delicatula*) |
|  |  | Coccidae | *Didesmococcus koreanus* Borchsenius (*D*. *koreanus*) | *D*. *koreanus* (*D*. *koreanus*) |
|  |  | Margarodidae | *Drosicha contrahens* (*D*. *contrahens*) | *D*. *contrahens* (*D*. *contrahens*) |
|  |  | Diaspididae | *Pseudacaspis pentagona* (*P*. *pentagona*) | *P*. *pentagona* (*P*. *pentagona*) |
|  |  | Aleyrodidae | *Bemisia tabaci* (Gennadius) (－) | *B*. *tabaci* (－) |
|  | True bugs | Pentatomidae | *Halyomorpha picus* (Fabricius), *Dolycoris baccaram* (Linnaeus), *Erthesina full* (Thunberg) (*H*. *picus*, *D*. *baccaram*, *E*. *full*) | *H*. *picus*, *D*. *baccaram*, *E*. *full* (*H*. *picus*, *D*. *baccaram*, *E*. *full*) |
|  |  | Miridae | *Adelphocoris fasciaticollis* Reuter, *Apolygus lucorum* (Meyer-Dur) (*A*. *fasciaticollis*, *A*. *lucorum*) | *A*. *fasciaticollis*, *A*. *lucorum* (*A*. *fasciaticollis*, *A*. *lucorum*) |
|  |  | Tingidae | *Stephanitis nashi* Esaki et Takeya (*S*. *nashi*) | *S*. *nashi* (*S*. *nashi*) |
|  |  | Coreidae | *Riptortus linearis* Fabricius (－) | *R*. *linearis* (－) |
|  | Orthoptera | Acrididae | *Atractomorpha sinensis* Bolivar, *Acrida cinerea* (*A*. *sinensis*) | *A*. *sinensis*, *A*. *cinerea* (*A*. *sinensis*) |
|  | Acarina | Tetranychidae | *Tetranychus viennensis* zacher, *Tetranychus cinabarinus*, *Tetranychus urticae* (*T*. *viennensis*, *T*. *cinabarinus*, *T*. *urticae*) | *T*. *viennensis*, *T*. *cinabarinus*, *T*. *urticae* (*T*. *viennensis*, *T*. *cinabarinus*, *T*. *urticae*) |
| Predatory arthropod | Coleoptera | Coccinellidae | *Leis axyridis* (Pallas), *Propylaea japonica* Thunbery, *Chilocorus kuwanae* Silvestri, *Coccinella septempunctata* (*L*. *axyridis*, *P*. *japonica*, *C*. *kuwanae*, *C*. *septempunctata*) | *L*. *axyridis*, *P*. *japonica*, *C*. *kuwanae*, *C*. *septempunctata* (*L*. *axyridis*, *P*. *japonica*, *C*. *kuwanae*, *C*. *septempunctata*) |
|  |  | Carabidae | *Carabus sp.* (*C*. sp*.*) | *C*. sp*.* (*C*. sp) |
|  | Neuroptera | Chrysopidae | *Chrysopa sinica* Tieder, *Sympetrum Croceolum*, *Chrysopa formosa* Brauer (*C*. *sinica*, *S*. *Croceolum*, *C*. *formosa*) | *C*. *sinica*, *S*. *Croceolum*, *C*. *formosa* (*C*. *sinica*, *S*. *Croceolum*, *C*. *formosa*) |
|  | Hemiptera | Anthocoridae | *Oriu minuius* Linnaeus (*O*. *minuius*) | *O*. *minuius* (*O*. *minuius*) |
|  | Diptera | Syrphidae | *Metasyrphus corollae*, *Baccha* sp. (*M*. *corollae*) | *M*. *corollae*, *Baccha* sp. (*M*. *corollae*) |
|  |  | Tachinidae | *Compsilura* sp*.* (－) | *Compsilura* sp*.* (－) |
|  | Mantedea | Mantidae | *Hierodula saussurei* Kirby (*H. saussurei*) | *H. saussurei* (*H. saussurei*) |
|  | Araneae | Thomisidae | *Misumenops tricuspidatus, Thomisus* sp*.* (*M*. *tricuspidatus, Thomisus* sp*.*) | *M*. *tricuspidatus, Thomisus* sp*.* (*M*. *tricuspidatus, Thomisus* sp*.*) |
|  |  | Erigonidae | *Erigonidium graminicola* (*E*. *graminicola*) | *E*. *graminicola* (*E*. *graminicola*) |
|  |  | Salticidae | *Myrmarachne* sp*.* (－) | *M*yrmarachne sp*.* (－) |
|  |  | Araneidae | *Argiope amoena*, *Neoscon,doenitzi*, *Argiope bruennichi*, *Araneus ventricosus*, *Neoscona* sp. (*A*. *amoena*, *N*. *doenitzi*, *A*. *bruennichi*, *A*. *ventricosus*) | *A*. *amoena*, *N*.*,doenitzi*, *A*. *bruennichi*, *A*. *ventricosus*, *Neoscona* sp*.* (*A*. *amoena*, *N*. *doenitzi*, *A*. *bruennichi*, *A*. *ventricosus*) |
|  |  | Lycosidae | *Lycosa pseudoamulata*, *Lycosa* sp. (*L*. *pseudoamulata*) | *L*. *pseudoamulata* (*L*. *pseudoamulata*) |
|  |  | Thomisidae | *Misumenopos tricuspidata* (Fahricius) (*M*. *tricuspidata*) | *M*. *tricuspidata* (*M*. *tricuspidata*) |
|  |  | Tetragnathidae | *Tetragnatha* sp. (*Tetragnatha* sp.) | *Tetragnatha* sp. (*Tetragnatha* sp.) |
|  |  | Dictynidae | *Dictyna felis* Boes. et Str. (*D*. *felis*) | *D*. *felis* (*D*. *felis*) |
|  |  | Theridiidae | *Theridion octomaculatum* Boes. et Str. (*T*. *octomaculatum*) | *T*. *octomaculatum* (*T*. *octomaculatum*) |
|  |  | Oxyopidae | *Oxyopes sertatus* (－) | *Oxyopes sertatus* (－) |

*Notes:* “－” means that there’s no corresponding species. The species outside and inside the parentheses were for peach orchards with and without ground cover *T*. *repens* respectively.
